# Are passive smoking, air pollution and obesity a greater mortality risk than major radiation incidents?

**DOI:** 10.1186/1471-2458-7-49

**Published:** 2007-04-03

**Authors:** Jim T Smith

**Affiliations:** 1 Centre for Ecology and Hydrology, Winfrith Technology Centre, Dorchester, Dorset DT2 8ZD, UK

## Abstract

**Background:**

Following a nuclear incident, the communication and perception of radiation risk becomes a (perhaps *the*) major public health issue. In response to such incidents it is therefore crucial to communicate radiation health risks in the context of other more common environmental and lifestyle risk factors. This study compares the risk of mortality from past radiation exposures (to people who survived the Hiroshima and Nagasaki atomic bombs and those exposed after the Chernobyl accident) with risks arising from air pollution, obesity and passive and active smoking.

**Methods:**

A comparative assessment of mortality risks from ionising radiation was carried out by estimating radiation risks for realistic exposure scenarios and assessing those risks in comparison with risks from air pollution, obesity and passive and active smoking.

**Results:**

The mortality risk to populations exposed to radiation from the Chernobyl accident may be no higher than that for other more common risk factors such as air pollution or passive smoking. Radiation exposures experienced by the most exposed group of survivors of Hiroshima and Nagasaki led to an average loss of life expectancy significantly lower than that caused by severe obesity or active smoking.

**Conclusion:**

Population-averaged risks from exposures following major radiation incidents are clearly significant, but may be no greater than those from other much more common environmental and lifestyle factors. This comparative analysis, whilst highlighting inevitable uncertainties in risk quantification and comparison, helps place the potential consequences of radiation exposures in the context of other public health risks.

## Background

Uncontrolled releases of radioactive material to the environment have major public health consequences over and above the direct health impacts of the radiation. For example, the economic, social and health impacts of the 1986 Chernobyl accident have been shown to have been greatly exacerbated by people's understandable fear of radiation [1,2]. The primary way of communicating unfamiliar risks to the public is by comparison with other more common risk factors. The present work carries out a novel assessment of radiation risk by evaluating scenarios for mortality risks from radiation and comparing these risks with risks from air pollution [3], obesity [4] and passive [5] and active [6] smoking.

It is always important to highlight the limitations of assessments of public health risk factors. The risk estimates presented here represent population-averaged increased mortality risks which cannot necessarily be interpreted as risks to the individual. Despite advances in the epidemiology of many health risk factors, direct quantification of different risks is still subject to significant uncertainty. For example, the timing and nature of a health detriment following a radiation exposure is likely to be different to that following exposure to air pollution. A recent study of health detriments from mercury in fish [7] has presented a risk-benefit analysis based on a "quality adjusted life years" approach which attempts to account for different timings and types of health detriment. Such an analysis is, however, beyond the scope of this paper as significant uncertainties remain in morbidity endpoints in some of the risk factors studied.

It is further noted that quantitative risk comparison is only one of many factors determining attitudes to risk [8] and that such comparisons cannot address some important ethical issues concerning, for example, differences between an imposed risk (radiation exposure in an extreme event) and a (to a certain extent) voluntary risk such as active smoking. A discussion of ethical issues related to radiation risk can be found in, for example, Oughton [9].

Extremely high doses of radiation lead rapidly to acute health effects (Acute Radiation Syndrome or ARS) which can be fatal. Many of the approximately 210,000 people who died in the immediate aftermath of Hiroshima and Nagasaki were victims of ARS and, following Chernobyl, 134 plant operators and emergency workers were diagnosed with ARS, 40 of whom died [10]. Lower, more prolonged, exposures to radiation do not necessarily lead to adverse health effects, but they can lead to an increased probability of a health detriment in later life. Because of their random nature, these effects are termed "stochastic" effects. Most importantly, following radiation exposure there is a certain probability that the individual will contract cancer in later life, though in most cases the exposure will have no effect. This paper focuses on cancer mortality risk and loss of life expectancy from ionising radiation and does not aim to give a full review of the health consequences of the Chernobyl accident and the Hiroshima and Nagasaki atomic bombs. Health consequences of Chernobyl (including, for example, ARS and thyroid cancer) have been reviewed elsewhere (e.g. [2,10-12]).

The scope of this paper is therefore to quantify different risks with their attendant uncertainties and differing health endpoints, with the focus here on mortality. The complex task of interpreting these risks and making (often subjective) value judgements on risk acceptability and risk comparison is beyond the scope of this paper.

## Methods

### Developing radiation risk scenarios

**Figure 1:**
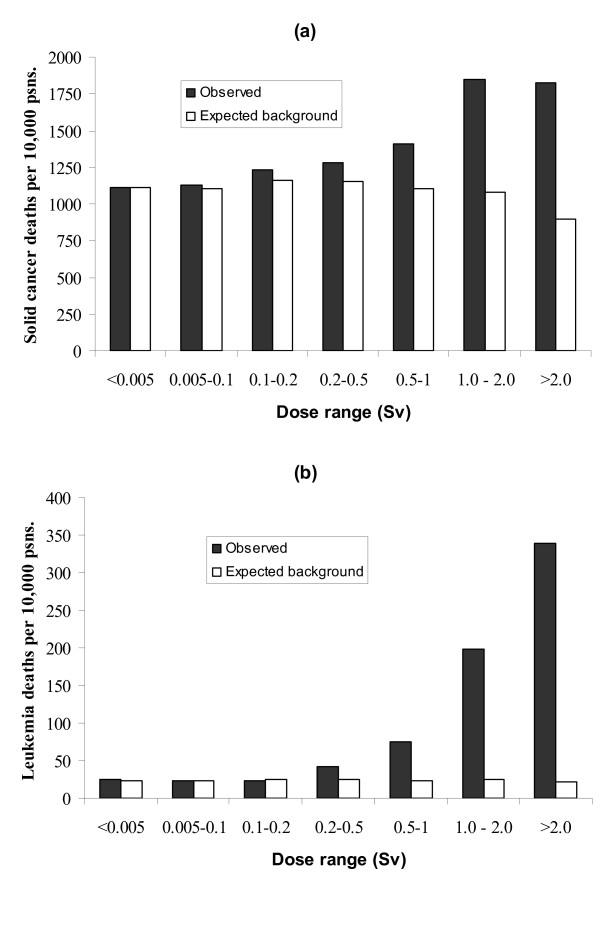
**Illustration of cancer mortality risk from ionizing radiation**. Fatal (a) solid cancer; (b) leukaemia rates (1950–2000) in people exposed to radiation from the Hiroshima and Nagasaki atomic bombs using data presented in ref. [13]. Mortality rates are per 10,000 people. In the 86,611 member cohort, of the 6061 cancer deaths observed in all persons exposed to more than 0.005 Sv, approximately 578 were attributed to radiation exposure.

Using epidemiological studies, primarily (but not only) of survivors of the Hiroshima and Nagasaki atomic bombs (Figure 1; [13]), radiation protection agencies have estimated the lifetime cancer risk to people from exposure to ionizing radiation [14,15]. Risk estimates recommended by the International Commission on Radiological Protection [15] are used to calculate stochastic radiation risks. These estimates predict a fatal cancer risk of 0.05 per sievert (Sv) of effective dose to the general population and 0.04 per Sv to the working population (the different population age distribution accounts for the difference in risk). The ICRP risk estimate implies, for example, that if a population is exposed to low dose rate radiation leading to an average effective dose equivalent of 0.1 Sv (100 mSv) to each person, an additional 0.5% of people will suffer a fatal cancer. Typically, the "natural" cancer incidence in industrialised countries is 20–25%. The radiation-induced cancers would not occur immediately, but may arise many years after exposure. Note that risks averaged over a population are presented here: the distribution of risks *within *a population will vary according to factors such as age and sex.

The ICRP risk estimate [15] assumes a dose and dose-rate effectiveness factor (DDREF) of 2.0 (reducing predicted risk by a factor of 2.0) for extrapolation of the data from the bomb survivors (who were exposed at extremely high dose rate) to lower dose and/or dose-rate exposures. "Low dose" has been defined as < 100 mSv [14], though there is no precise definition. Some assessments of cancer mortality following Chernobyl (e.g. [11]) did not apply a DDREF, whilst others did (e.g. [16]). The present study uses the ICRP risk estimates which include a DDREF, but where appropriate it is noted that risk predictions would be increased by a factor of 2 should the DDREF be excluded.

In calculating radiation risks, the ICRP approach [15] has been used for consistency. Separate risk factors were applied for exposures to the average population and for occupational exposures to the population of working age. The US National Academy of Sciences has also recently re-assessed risks from low dose, low linear energy transfer radiation [14], in particular updating uncertainty estimates in risk. These new risk estimates were compared with the previous ICRP [15] estimates and no substantial differences were found for the cases studied here. It is further noted that the US National Academy of Sciences [17] and US Environmental Protection Agency [18] have recently re-assessed the lung cancer risk from exposures to radon in the home. This risk estimate is discussed further below.

For the inter-comparison of radiation risks, scenarios were chosen in order to illustrate a range of different exposures from a few mSv up to several hundred mSv. For the comparison of radiation risks with other environmental risk factors, doses to approximately 200,000 Chernobyl emergency workers ("liquidators") were used as an illustration of relatively high exposures after a radiation incident. As exposures to passive smoking and air pollution represent averages for the exposed group it is appropriate to compare these with average exposures to the Chernobyl emergency workers (100 mSv). But exposures to the high dose group (250 mSv) of Chernobyl emergency workers are also presented for comparison. For comparison of radiation risks with active smoking and obesity, loss of life expectancy of atomic-bomb survivors in the high dose group (2.25 Gy) is used. Lower exposures led to a significantly lower loss of life expectancy: for example, those exposed to < 1 Gy (mean 140 mGy) had a life expectancy which was on average 70 days shorter than that of zero-dose individuals [19].

Radiation exposures were estimated for each of the illustrative radiation risk scenarios. The percentage mortality from low dose/dose rate radiation was calculated using:

% *mortality risk *= 100.*H*_*E*_.*R*_*c*_

where *H*_*E *_(in Sv) is the effective dose, and *R*_*c *_(in Sv^-1^) is the ICRP [15] risk coefficient for either the general or working-age populations.

For long term exposures from the Chernobyl accident (primarily from ^137^Cs), an effective ecological half life of 25 years was conservatively assumed [20] to model the change in exposure over time. For example, in the scenario illustrating consumption of sheep meat in the UK, doses to "critical group" consumers were estimated for a "worst case" scenario of exposure to an infant born in 1986 consuming lamb from affected farms for his or her 75 year lifetime. Critical group intake rates and effective doses per unit intake were taken from [21]. It was assumed that lamb was contaminated with 500 Bq kg^-1 ^in 1986 declining with effective ecological half life 25 years [20]. This is an over-estimate of likely real exposures.

### Risks from air pollution

A recent cohort study of approximately 500 000 adults in US cities [3] showed that "each 10 μg m^-3 ^elevation in fine particulate [PM_2.5_, particles less than 2.5 μm diameter] air pollution was associated with approximately a 4%, 6%, and 8% increased risk of all-cause, cardiopulmonary, and lung cancer mortality, respectively". The relative risk (RR) of all cause mortality was 1.04 with CI 1.01–1.08. It is assumed that the epidemiological findings of Pope et al. [3] indeed represent causal relationships between ambient air pollution (as measured by PM_2.5_) and mortality.

In 2005, the mean annual PM_10 _concentration in London was approximately 28.5 μg m^-3 ^(mean of 8 sites) compared to 17 μg m^-3 ^in Inverness [22], the least polluted of the UK cities monitored by DEFRA. Using a ratio PM_10_:PM_2.5 _= 1.67 applied to US data [23] (c.f. a study in Birmingham (UK) [24] which gave a ratio of means of 1.61) this gives estimated PM_2.5 _of 17.1 μg m^-3 ^and 10.2 μg m^-3 ^in London and Inverness respectively. (Note also that a study reported in [23] measured a mean value of 18 μg m^-3 ^PM_2.5 _at 3 urban background sites in London in 2000–2001). If the Pope et al. [3] finding of 4% increased mortality risk per 10 μg m^-3 ^increase in PM_2.5 _is applicable to the UK, this difference represents a predicted 2.8% increase in mortality in Central London (compared to Inverness) as a result of air pollution.

### Risks from obesity

It is well known that increased body fat can lead to increased risk of mortality. An individual's body fat is usually defined by their body-mass index (BMI, kg/m^2^). It is now well known that high BMI potentially poses major health risks to a significant proportion of people in developed countries. For example, 127 million Americans are classed as overweight (25.0 ≤ BMI ≤ 29.9), 60 million are classed as obese (30.0 ≤ BMI ≤ 39.9) and 9 million are classed as severely obese (BMI ≥ 40) [25].

**Figure 2:**
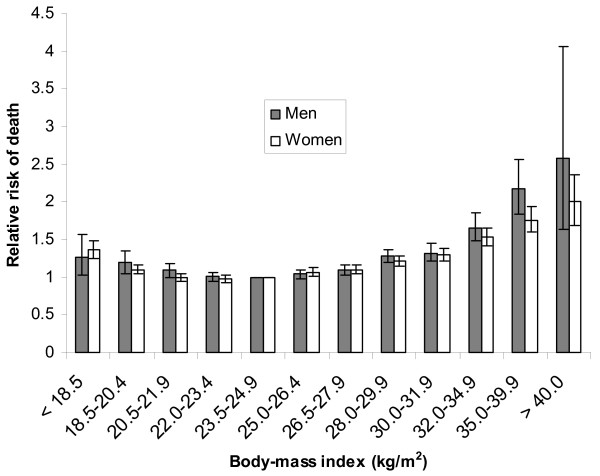
**Illustration of mortality risk vs Body Mass Index**. Relative risk of mortality vs Body Mass Index in white, non-smoking, men and women (from data in ref. [4]). Error bars show the 95% CI in relative risk.

A study of more than 1 million US adults [4] analysed the relationship between BMI and mortality in a 300,000 person sub-group of non-smokers. The study adjusted for other potential risk factors such as level of education and physical activity. Whilst noting some remaining uncertainties [4], the study of this sub-group showed a clear relationship between BMI and all-cause mortality, and mortality from both cardiovascular disease and cancer (Figure 2). Fontaine and coworkers [26] used data from the U.S. National Health and Nutrition Examination Surveys to determine years of life lost (YOLL) due to overweight and obesity in comparison with a reference BMI of 24.

Note that a recent study [27] observed only increased mortality (relative to a 18.5 < BMI < 25.0 control group) in groups with BMI > 30.0 and decreased relative mortality in a group with BMI between 25 and 29.9 (overweight). This has caused controversy over the BMI-mortality relationship: the Flegal et al. [27] study contradicts earlier studies, but has been criticised by other workers in the field (for both sides of this discussion, see [28,29]).

### Risks from smoking and passive smoking

**Figure 3:**
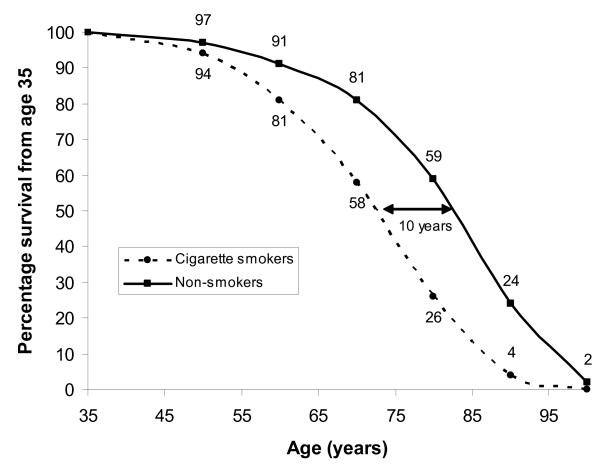
**Illustration of mortality risk from active smoking**. Predicted survival curve from age 35 for smoking and non-smoking male doctors (reproduced from data presented in [6] with permission from the BMJ Publishing Group). Percentage of original population surviving is shown at each decade.

About half of all smokers suffer an early death from a smoking-related disease [6]. The relationship between smoking and a number of different cancers [30] and cardiovascular diseases [31] is of course well established. For example, in a 50-year study of health effects of smoking in male British doctors [6], it was shown that a 35 year old male doctor who smoked had a life expectancy approximately 10 years lower than for those doctors who had never smoked (Figure 3).

Because of the much lower relative risks involved, levels of adverse health effects from passive smoking are less certain than those from active smoking. Studies have, however, shown correlations between passive smoking and a number of diseases including lung cancer and heart disease. A meta-analysis of 37 epidemiological studies of lung cancer [32] found a relative risk (RR) of lung cancer of 1.24 (CI: 1.13–1.36) in non smokers who lived with a smoker compared to non-smokers whose partner did not smoke. A meta-analysis of 19 published studies [33] observed a relative risk of heart disease of 1.23 (CI: 1.14–1.33) in non-smokers who lived with a smoker compared to non-smokers whose partner did not smoke. A number of other potentially fatal conditions have also been linked to passive smoking [34].

It has been estimated that in the U.S., passive smoking annually accounts for 60,460 excess deaths comprised of 47,000 deaths from heart disease, 3,060 deaths from lung cancer and 10,400 from other cancers [35]. A study [36] of ischaemic heart disease (IHD) estimated an excess mortality risk for a non-smoker living with a spouse who smokes of 2.2% for men and 1.2% for women (assumed RR for heart disease: 1.31 and 1.24 respectively).

## Results and discussion

### Exposure and risk scenarios

Different radiation risks can be directly compared to each other since there is a common link between exposure and risk. Such risk comparisons are based on the LNT model, combined with internal and external exposure models and estimates of the biological effectiveness of different radiation types. Table 1 summarises different exposures and risks from natural and medical radiation sources and compares these with illustrative exposures following the Chernobyl accident. Doses are expressed as risk for the total exposure over the specified time period. Note that exposures from Chernobyl do not here include dose to the thyroid or the 134 cases of ARS resulting from exposures during the accident (for a summary of the health effects of Chernobyl, including thyroid cancer, see [2,10-12]).

**Table 1:** Illustrative radiation exposures from natural background, medical, routine nuclear operations and Chernobyl with hypothetical lifetime risks.

**Exposure scenario**	**Exposure**	**Mortality risk**^+^	**Notes**
***Examples of radiation exposures not due to Chernobyl***			
*General population (background exposures)*			

UK average (natural + medical)	200 mSv	**1 %**	Lifetime (~75 yr) exposure to 2.7 mSv yr^-1 ^UK average annual dose.
Exposure at UK limit for radon exposures in the home [49]	750 mSv	**3.7 %**	Lifetime (~75 yr) exposure to UK limit 200 Bq m^-3 ^radon gas ≈ 10 mSv yr^-1 ^dose. Above this limit, action must be taken to reduce radon in houses in the UK. Dose depends on time spent at home and doses at this high rate are rare.

Working population (above background)*			

UK average for classified radiation workers [50]	18 mSv	**0.07%**	Average current dose (above background) of classified workers in the nuclear industry of 0.6 mSv yr^-1 ^accumulated over a 30 year working period.
Long haul air crew [51]	135 mSv	**0.54%**	Typical exposures in the range 3–6 mSv yr^-1^: assume 4.5 mSv yr^-1 ^over 30 yrs

Exposures after Chernobyl (above background)*			
*General population*			

Residents of "strict control zones" (areas > 555 kBq m^-2 ^^137^Cs).	50 mSv	**0.25%**	Accumulated dose for approximately 10 year period after the accident [37, 52]
Annual dose limit to populations of the Chernobyl affected areas, 1990's	75 mSv	**0.37%**	If external + internal dose exceeded this limit, measures had to be taken to reduce dose. Accumulated dose at 1 mSv yr^-1 ^over 75 yr lifetime [53]
Consumer of sheep meat from the most contaminated areas in the UK	4.1 mSv	**0.02%**	Consumption (at a high rate) of lamb from farms most affected by Chernobyl for 75 year period (assumed mean ^137^Cs = 500 Bq kg^-1 ^in 1986, declining with effective half life 25 yr). Over-estimate of likely real exposures.

*Working population*			

Unofficial residents of the 30-km exclusion zone. In late 1990's range of doses in a number of villages [2], Ukrainian sector 30 km zone was 1–6 mSv y^-1^	255 mSv	**1.0%**	Illustrative of higher exposures: person of working age (25) who received 100 mSv during period to 1995, then returned to Zone in 1996 and received 6 mSv yr^-1 ^in 1996 declining (with effective half life 25 years) to age 75 in 2036. N.B. some (uninhabited) areas of the Zone would give much higher doses.
Chernobyl emergency workers [37]: Average High dose group	100 mSv 250 mSv	**0.4%** **1.0%**	Accumulated risk from exposures during 1986–87. Does not include very high exposures to those who suffered from ARS. Working population.

+ A DDREF of 2 is applied for these (relative to the Japanese bomb survivors) low dose rate exposures. If the DDREF were not used all the risk factors would increase by a factor of 2. * These exposures are in addition to those from background radiation. Note that exposures to the 134 ARS victims and doses to the thyroid following Chernobyl are not included here (see text).

It is clear from Table 1 that current exposures from the Chernobyl accident are not greater (and are in some cases much smaller) than some exposures to natural background radiation (e.g. long-haul air crew or some residents of relatively high natural background areas). Doses to the population of approximately 200,000 emergency workers who worked in the Chernobyl 30-km exclusion zone in 1986–87 averaged approximately 100 mSv and doses to residents of the "strict control zones" were on average lower, but of the same order [37]. A group of people living unofficially in the 30-km exclusion zone around Chernobyl were found to receive annual doses of 1–6 mSv yr^-1 ^in the late 1990s [2]. A lifetime's exposure to high natural background radiation in some parts of the world can result in an accumulated dose of 700 mSv or more (Table 1). More than 100,000 people in Finland, for example, receive natural radiation doses > 10 mSv yr^-1 ^[10].

Mortality risks from example exposure scenarios for air pollution, passive smoking and radiation are shown in Table 2. Comparing risks between different risk factors is more uncertain than comparisons between different radiation sources. The time delay in the health impact following exposure to passive smoking or air pollution, for example, may be different to that following exposure to low-dose radiation.

**Table 2:** Approximate hypothetical lifetime increased mortality rate from illustrative scenarios of exposure to air pollution, passive smoking and radiation^a^.

**Exposure scenario**	**Exposure**	**Health endpoint**	**Approximate lifetime increased mortality**
Living in Central London compared to Inverness.	Mix of air pollutants indicated by average PM_2.5 _= 6.9 μg m^-3 ^higher.	Mortality	**2.8 %** Postulated 2.8% higher air pollution related mortality in central London compared to Inverness (see text).
N.B. Extrapolates from data in the US. May be confounding factors which, if accounted for, would change the excess risk. Time-lag between exposure and effect is uncertain.			

Passive smoking – risk to non-smoker at home if spouse smokes.	Mix of pollutants in secondhand smoke.	Mortality	**1.7 %** 1.7% lifetime excess IHD mortality risk from passive smoking: average for men and women [36].
N.B. Heart disease risk: does not include strokes or the (significantly lower) risk from lung cancer or other illnesses. May be confounding factors/limitations of meta-analysis data.			

Chernobyl emergency workers in the 30-km Zone 1986–87.	Radiation exposure: 100 mSv 250 mSv Illustrative of mean (100 mSv) and high (250 mSv) doses: 4% of workers received doses >250 mSv.	Mortality	**0.4 %** **1.0 %** Predicted 4% risk of fatal cancer for 1000 mSv dose to working age population.
N.B. Uncertainty in extrapolation from high dose and dose rate Japanese data to these chronic low doses. If the DDREF was not applied, mortality risk would increase by a factor of 2. Time lag between exposure and effect is generally long (> 10 years) for solid cancers, but is shorter (< 15 years) for leukaemia. Note that 134 ARS victims received much higher doses than 250 mSv.			

a. Note that health impacts change (generally, but not always, increase) with age. Risk also varies with age at time of exposure. For example, for air pollution, risks are believed to be higher for older people, but for radiation risks are higher from exposure at a young age (though effects may be observed after a long latency period). Risks may be distributed within the population in a different way for different risk factors. All risk factors have potential impacts on morbidity (illness) in addition to mortality.

There are significant uncertainties in risks in all the cases shown in Table 2, however, this comparison of time- and population-averaged risks can help to put radiation risks in context. The radiation exposures to emergency workers and to the most exposed populations following Chernobyl represented a potentially significant increase in fatal cancers in the exposed populations. But, the risk (from the evidence analysed here) appears to be no greater than potential mortality risks from air pollution, passive smoking, or high natural background radiation exposures.

Table 3 compares risks from acute, high dose radiation with active smoking and high BMI, in terms of expected average reduction in lifespan. Both of these latter risk factors are to a large extent determined by individual choice, though both are also influenced by cultural and socio-economic conditions. Active smoking and BMI therefore provide quantitative risk comparators for acute high dose radiation exposure. However, there is no intention here to make an ethical comparison between an imposed risk (radiation exposure in an extreme event) and a (to an extent) voluntary risk such as smoking or high BMI.

**Table 3:** Loss of life expectancy due to smoking, high body mass index and the long term effects of high acute radiation exposure.

**Risk scenario**	**Average Years of Life Lost (YOLL)**	**Notes**
**Smoking** Male doctor who is a lifetime smoker compared to non-smoker.	10	Ref. [6]. Average smoking habit: 18 a day from age 18.

**Obesity** White male aged 35 who is obese (BMI = 30.0–39.9) or severely obese (BMI >40): risk relative to BMI = 24.	Obese: 1–4 ^a^ Severely obese: 4–10^a^	Ref. [26]. There is controversy over the BMI-mortality relationship (see text). However, increased mortality at BMI > 30 has been observed in a number of studies, though there is uncertainty in excess mortality rate and hence YOLL.

**Radiation** Atomic bomb survivor who was in the most exposed group: within 1500 metres of the hypocentre. Shielded whole body kerma > 1 Gy, mean 2.25 Gy.	2.6 (1.3–5.2)^a^	Ref. [19]. Only represents YOLL of bomb survivors. Few people close to the hypocentre survived the combination of blast effects, burns and ARS.

a. Ranges are for different BMI or dose rates and are not uncertainty estimates.

The comparison for extreme radiation risks in Table 3 may be of limited value since such exposures are, fortunately, rare. In addition, the comparison does not account for the deterministic (i.e. ARS) effects of acute exposures in the range 1–5 Gy which (by definition) does not influence the YOLL of these A-bomb survivors. However, Table 3 does put the health risks of active smoking and obesity into a novel perspective.

### Radiation risks

The risk estimates recommended by the International Commission on Radiological Protection [15] are for chronic exposures at relatively low dose rate rather than the high dose rate exposures to the atomic bomb survivors. In radiation risk assessments it is current practice to assume that even very low dose radiation carries with it an associated cancer risk (the linear, no-threshold or LNT model). This assumption is based on radiobiological evidence that DNA damage from a single radiation impact can potentially lead to cancer. Although often inconclusive at very low doses, epidemiological evidence also tends to support the LNT model. A recent study [38] has shown statistically significant excess cancer risk at acute doses down to 60 mSv in the Japanese bomb survivors. In a review [39] which included studies of medical and occupational radiation exposures, it was argued that "good evidence of an increase in risk for cancer is shown at acute doses > 50 mSv, and reasonable evidence for an increase in some cancer risks at doses above ≈ 5 mSv... good evidence of an increase in some cancer risks is shown for protracted ["chronic"] doses > 100 mSv, and reasonable evidence ... at protracted doses above ≈ 50 mSv".

Exposure to low level radiation can potentially result in hereditary effects on subsequent generations. Evidence of effects on offspring has been observed in studies on laboratory animals [40]. Studies on the children of the survivors of the Hiroshima and Nagasaki bombs have, however, found no evidence of hereditary effects of radiation [41].

Lung cancer from exposures to radon and its decay products forms the major excess risk at high radon concentrations in the home. The US National Academy of Sciences [17] and US Environmental Protection Agency [18] have recently re-assessed the lung cancer risk from exposures to radon in the home. The stochastic mortality risk of 3.7% at lifetime radon exposure of 750 mSv (Table 3, as calculated from [15]) will therefore be compared with these more recent radon risk estimates.

The lifetime fatal lung cancer risk to an average member of the US population at an average radon air concentration of 37 Bq m^-3 ^is 0.58% assuming 70% of time is spent at home [18]. At the UK action level for radon in the home (200 Bq m^-3^), assuming LNT, this corresponds to a lifetime fatal lung cancer risk of 3.1%. This compares well with the mortality risk estimate of 3.7% presented in Table 1 for lifetime radon exposure at the UK action level, though this does not necessarily imply that the ICRP and EPA risk coefficients are the same: the former risk is calculated on the basis of an estimated effective radiation dose whilst the latter relates risk directly to radon concentration in air from epidemiological studies of miners. In addition, it should be noted that the more recent radon risk estimates [17,18] show a much higher excess absolute risk in smokers than in non-smokers due to the synergistic effects of smoking and radon. The risk estimate presented here is for an average population of smokers and non-smokers (as is the case in the ICRP approach).

### Air pollution risks – time series vs. cohort studies

It is well known that air pollution in cities can lead to significant health problems. The London smog of 1952 was reported to have caused an extra 4000 deaths in the capital and a huge increase in hospital admissions for respiratory and cardiovascular diseases. A pollution episode in December 1991 was associated with an additional 101 to 178 deaths in London [42]. The impacts of air pollution on health may be estimated by studies of short-term relationships between incidents and immediate health effects ("time-series studies") or by "cohort" studies relating long-term air pollution to average morbidity (illness) and mortality rates.

Time-series studies have identified clear relationships between pollution episodes and mortality as exemplified by the London incidents. There is uncertainty, however, concerning assessment of the impact of such short-term incidents, particularly in assessing the years of life lost (YOLL) of the victims. Analyses of such incidents have shown that they tend to bring forward the deaths of elderly or seriously ill people (by a relatively small time period) rather than immediately affecting generally healthy people. A report of the Committee on the Medical Effects of Air Pollutants [23] assumed that the loss of life expectancy following short-term pollution episodes is on average in the range 2–6 months, though it is possible that deaths are brought forward by just a few days in many cases.

Longer-term cohort studies, on the other hand, tend to emphasise the long-term effects of chronic exposures. For example, the U.S. "Six Cities Study" [43] followed the health of a group of 8111 adults from 1974–1991. The mortality rate in the most polluted of the six cities was 1.26 times higher than in the least polluted city (95% CI: 1.08–1.47). Deaths from lung cancer and cardiopulmonary disease were correlated with levels of fine particulate air pollution.

A discussion of the differences between cohort and time-series studies of air pollution can be found in [44]. It has been suggested [23] that reductions in air pollution would lead to a "gain in life years from the cohort studies [which] is at least 10-fold greater than estimates from the time-series studies alone". Thus, cohort studies show a much greater influence of air pollution on YOLL than time-series studies. It has been noted [44] that "the total impact (YOLL) of air pollution advancing deaths by a long time ... is estimable from cohort studies results". The meaning of "a long time" in this context is not precisely defined, but is likely to be greater than several months [44].

Whilst noting the many uncertainties and potential confounding factors in cohort studies, these can be used to make tentative estimates of deaths brought forward by a "long time" as a result of exposure to air pollution.

### Uncertainties

All of the risk estimates discussed above are based on epidemiological studies and are therefore subject to statistical uncertainties and potential confounding factors. Quoted confidence intervals are limited in that they do not necessarily encapsulate all possible sources of error in relative risk estimates: it is rarely (if ever) possible to account for all confounding factors. The limitations of epidemiological studies are well known and results need to be treated with great caution, particularly when observed relative risks are low (less than, say, 2–3; [45]). Some of the risk factors discussed here (acute exposure to > ~100 mSv radiation, active smoking, very high BMI) are based on strong epidemiological evidence and show clear dose-response relationships, as illustrated in Figures 1, 2, 3. The other risk factors (chronic low-dose radiation, passive smoking, air pollution) are all subject to much greater uncertainty and potential bias.

For statistical analyses of the various epidemiological studies used, the reader is referred to the original references on which the excess relative risks are based. It is not always possible to present accurate objective confidence intervals for these risk estimates. Where possible, confidence intervals of relative risks are presented here, though accurate confidence intervals were not always available (for example, ref. [14] cites only a subjective CI). It is also noted that quoted confidence intervals are limited in that they do not necessarily encapsulate all possible sources of error in relative risk estimates. Uncertainties in the various risk factors are summarised in Table 4.

**Table 4:** Summary of available uncertainties in various risk factors.

**Risk factor**	**Uncertainty**
Air pollution: 10 μg m^-3 ^increase in PM_2.5_	*RR *of mortality is 1.04 with 95% CI: 1.01–1.08 [3] but note unexamined confounding factors could increase uncertainty.

Passive smoking: Long-term exposure compared to little or no exposure.	*RR *of lung cancer [32] is 1.24 with 95% CI: 1.13–1.36 *RR *of heart disease [33] is 1.23 with 95% CI: 1.14–1.33 Excess mortality risk [36] was based only on heart disease *RR *of 1.31 and 1.24 for males and females respectively, at the higher end of the range given by [33].

Obesity: High BMI compared to "normal" BMI = 24	Uncertainty in YOLL not presently available. Ref. [26] states that "we were unable to provide confidence intervals for our YLL estimates. We are unaware of any developed analytic formula that would allow easy calculation of SEs and confidence intervals". Uncertainties in relative risks are illustrated in Figure 2.

Radiation: Risk per unit dose equivalent.	Subjective 95% CI was given for NAS risk analysis [14] where it was stated that "estimates that are a factor of two or three larger or smaller cannot be excluded" (see also [54]). This uncertainty is expected to also apply to the ICRP [15] risk estimates presented here. In particular, it is uncertain whether a DDREF should be applied: if a DDREF was not applied, this would increase the ICRP risk estimates by a factor of 2.

The risks arising from chronic, low-dose radiation are determined to a large extent by linear extrapolation (LNT model) from the data on Japanese atomic bomb survivors, with a reduction due to predicted lower effectiveness of low dose rate radiation in cancer induction (the DDREF). There are ongoing arguments concerning the shape of the dose-response curve at low doses and dose rates with some arguing that risks may be significantly higher or lower than predicted by the standard extrapolation from high dose data.

There is also uncertainty in the risks of passive smoking and air pollution. Both air pollution and passive smoking studies may be compromised by socio-economic, environmental or lifestyle factors which could not be accounted for, even in large scale studies or meta-analyses [46-48]. In addition, cohort studies of air pollution are necessarily based on health risks from past (generally higher) exposures which may not apply today [46].

## Conclusion

Whilst acknowledging the inevitable uncertainties in risk assessment, the communication and mitigation of public health risks must be based on the best available scientific evidence. Nuclear incidents clearly have many serious consequences, a full review of which is beyond the scope of this paper. But the assessment of "best estimate" risk scenarios presented here provides a context within which to communicate the long-term mortality risk to those exposed to radiation following such incidents. Such risk communication could help to mitigate some of the serious social, economic and psychological impacts of incidents involving radiation. When considered in the context of other more common public health risk factors, the long-term mortality risks from radiation exposures following major incidents, whilst very serious, appear to be less serious than is commonly perceived. For example:

• The radiation exposures to the populations most affected by the Chernobyl accident (emergency workers and people continuing to live in contaminated areas) results in an average additional mortality risk no greater than that caused by (relatively common) elevated exposures to natural background radiation either at home or through occupation.

• The increased mortality rate of the populations most affected by the Chernobyl accident may be comparable to (and possibly lower than) risks from elevated exposure to air pollution or environmental tobacco smoke. It is probably surprising to many (not least the affected populations themselves) that people still living unofficially in the abandoned lands around Chernobyl may actually have a lower health risk from radiation than they would have if they were exposed to the air pollution health risk in a large city such as nearby Kiev.

• The immediate effects of the Hiroshima and Nagasaki atomic bombs led to approximately 210,000 deaths in the two cities. However, radiation exposures experienced by the most exposed group of survivors led to an average loss of life expectancy significantly lower than that caused by severe obesity or active smoking.

## Competing interests

The author(s) declare that they have no competing interests.

## Authors' contributions

JS carried out all aspects of study design and manuscript preparation.

## Pre-publication history

The pre-publication history for this paper can be accessed here:


